# Individualized prognostic signature for pancreatic carcinoma validated by integrating immune-related gene pairs (IRGPs)

**DOI:** 10.1080/21655979.2020.1860493

**Published:** 2021-01-04

**Authors:** Yecheng Li, Pingan Yao, Kui Zhao, Zhenyu Ye, Haobo Zhang, Jianping Cao, Shuyu Zhang, Chungen Xing

**Affiliations:** a Department of General Surgery, Second Affiliated Hospital of Soochow University, Souzhou Jiangsu, China; b School of Radiation Medicine and Protection and Jiangsu Provincial Key Laboratory of Radiation Medicine and Protection, Medical College of Soochow University, Suzhou, China; c Second Affiliated Hospital of Chengdu Medical College, China National Nuclear Corporation 416 Hospital, Chengdu, China

**Keywords:** IRGPs, pancreatic carcinoma, prognostic, bioinformatics, retrospective analysis

## Abstract

Increasingly attention is being given to immune molecules in pancreatic cancer. The purpose of this study was to understand the potential clinical application of immune-regulated genes (IRGs) in the stratification of prognosis and to facilitate the development of personalized prognostic information for pancreatic cancer patients. We systematically used public data to comprehensively analyze immune-regulated gene pair (IRGP) expression profiles and clinical data. In our study, IRGP signature was identified to predict the overall survival (OS) of pancreatic cancer patients. We suggested that immune genes are enriched in different risk groups. In the high-risk group, M1 macrophages and resting NK cells were significantly enriched, while the percentages of naïve B cells, resting dendritic cells, CD8 T cells and regulatory T cells (Tregs) were significantly higher in the low-risk group, and we verified these results with immunohistochemical experiments. Gene ontology (GO) analysis confirmed that the IRGP index (IRGPI) signature genes in the cohort were mostly party to sensory perception of a chemical stimulus and the adaptive immune response. The identification of these pathways provides a basis for studying the molecular mechanisms of IRGPI signaling to predict the prognosis of pancreatic cancer. Our study effectively constructed a robust IRGP signature with prognostic value for pancreatic cancer, presenting a conceivable method for deciding on a preoperative treatment.

## Graphical Abstract

**Figure:**
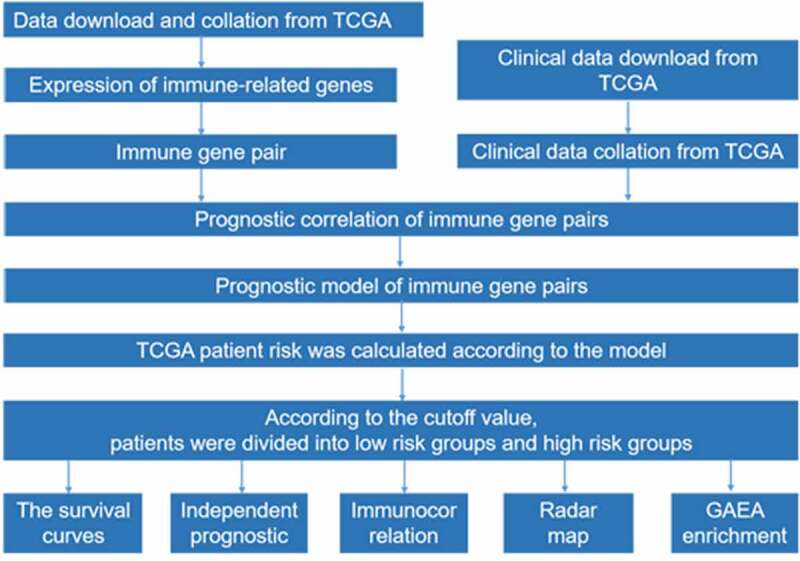

## Introduction

Pancreatic carcinoma is one of the deadliest gastrointestinal malignancies [1]. Pancreatic cancer causes 216,000 new cases and >200,000 deaths a year worldwide [2]. It has become the third most common cause of cancer-related death in America, and the prognosis varies among individuals [3]. Several factors, such as cigarette smoking, diabetes mellitus, chronic pancreatitis and obesity, can affect the risk of pancreatic carcinoma [4,5]. Early pancreatic carcinoma is commonly asymptomatic, and pancreatic cancer generally remains asymptomatic until it develops into advanced pancreatic cancer [6]. When the patient first developed symptoms, pancreatic carcinoma patients with stage 0 and I disease account for 0.7% and 2.3%, respectively, and three quarters of these patients have no symptoms [7]. Although improvements in treatment have been made, the 5-year survival rate of pancreatic cancer remains dismal at less than 6%, which is not satisfactory [8]. Tumor staging, histological grading, and molecular subtyping are used to evaluate prognostic factors in patients with pancreatic cancer in the clinic. However, these clinicopathological features usually do not provide information to accurately predict patient prognosis [9]. Some inflammatory molecules have been linked to the prognosis of pancreatic cancer, but their sensitivity and specificity are extremely variable [10]. These factors may cause inaccurate prognostic predictions for pancreatic cancer patients, and there is still no accurate indicator to predict the prognosis of pancreatic cancer. Therefore, to successfully treat pancreatic cancer patients, the identification of new and reliable molecular markers to predict the prognosis of pancreatic carcinoma patients is urgently needed.

Systemic therapy is still recommended, as the majority of pancreatic patients have micrometastatic disease at the time of diagnosis [9]. Numerous studies have shown that immunotherapies are one of the most exciting treatment strategies to emerge over the last decade[1]. For instance, there is evidence that PD-L1 can predict the prognosis of pancreatic cancer and be used as an effective target for the treatment of pancreatic cancer [11,12]. However, there are currently no targeted therapies for pancreatic cancer directed against the main known driver mutations [13]. The molecular characteristics of immune interactions require further study. However, there are problems such as too small fitting of small sample datasets and insufficient verification datasets may reduce the robustness and efficiency of conclusions [14,15]. A new technique primarily based on relative sequencing of gene expression levels is proposed, which eliminates the disadvantages of data normalization and scale transformation in gene expression data processing and has produced reliable results in quite a number researches [16–18].

Increasingly, attention is being given to immune molecules in pancreatic cancer [19]. At present, there are few studies on the screening and identification of molecular markers that can predict the prognosis of pancreatic cancer by means of mass immune-regulated gene (IRG) expression profiles. The purpose of this study was to understand the potential clinical application of IRGs in the stratification of prognosis and to facilitate the development of personalized prognostic information for pancreatic cancer patients. We systematically used public data to comprehensively analyze immune-regulated gene pair (IRGP) expression profiles and clinical data.

## Results

### IRGP signature construction and its prognostic valuation

**Figure 1.**
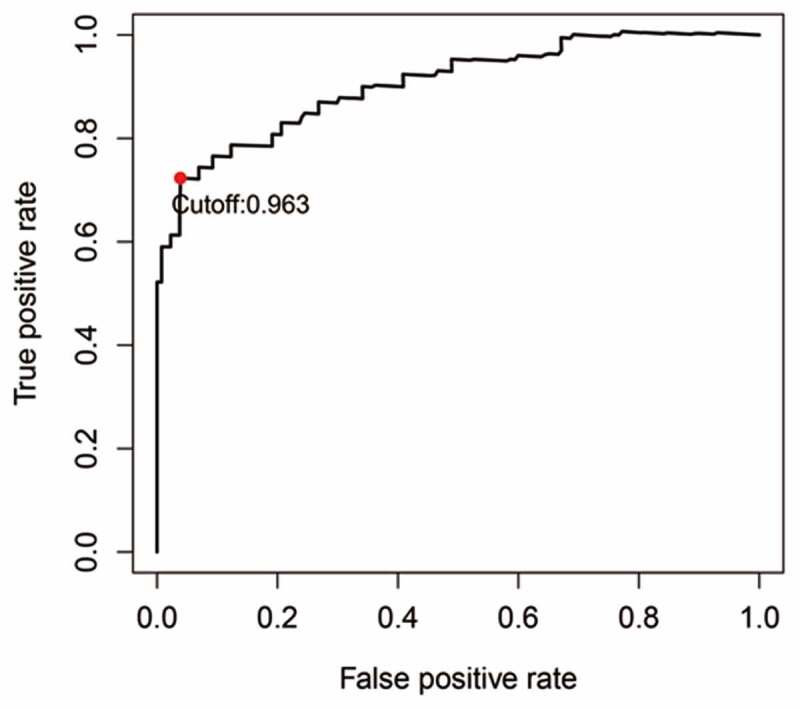
Time-dependent ROC curve for the IRGPI in the cohort. An IRGPI score of 0.963 was used as the cutoff for the IRGPI to stratify pancreatic cancer sufferers into low- and high-risk groups

**Figure 2.**
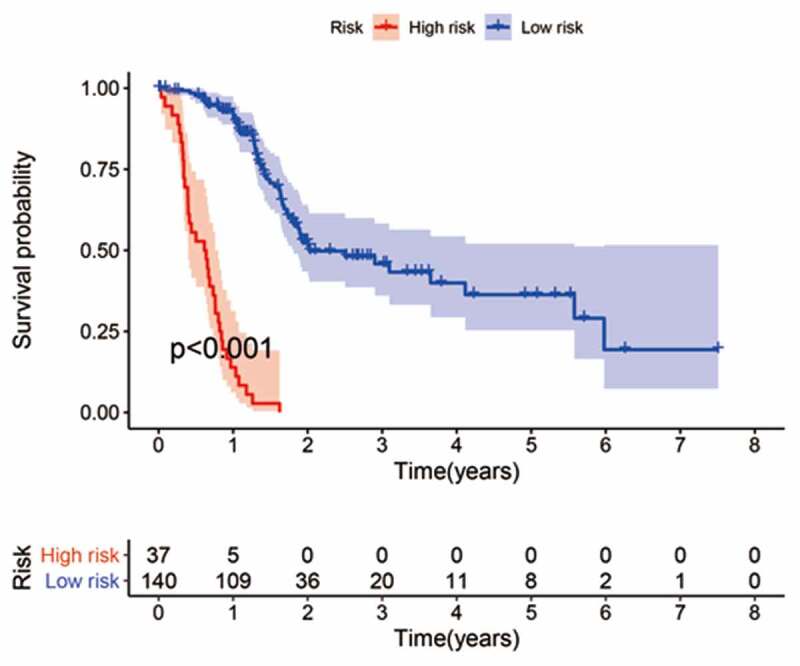
Kaplan-Meier curves for the overall survival (OS) of different IRGPI-defined risk groups. The OS of the high-risk group was significantly shorter than that of the low-risk group (*p* < 0.001)

**Figure 3.**
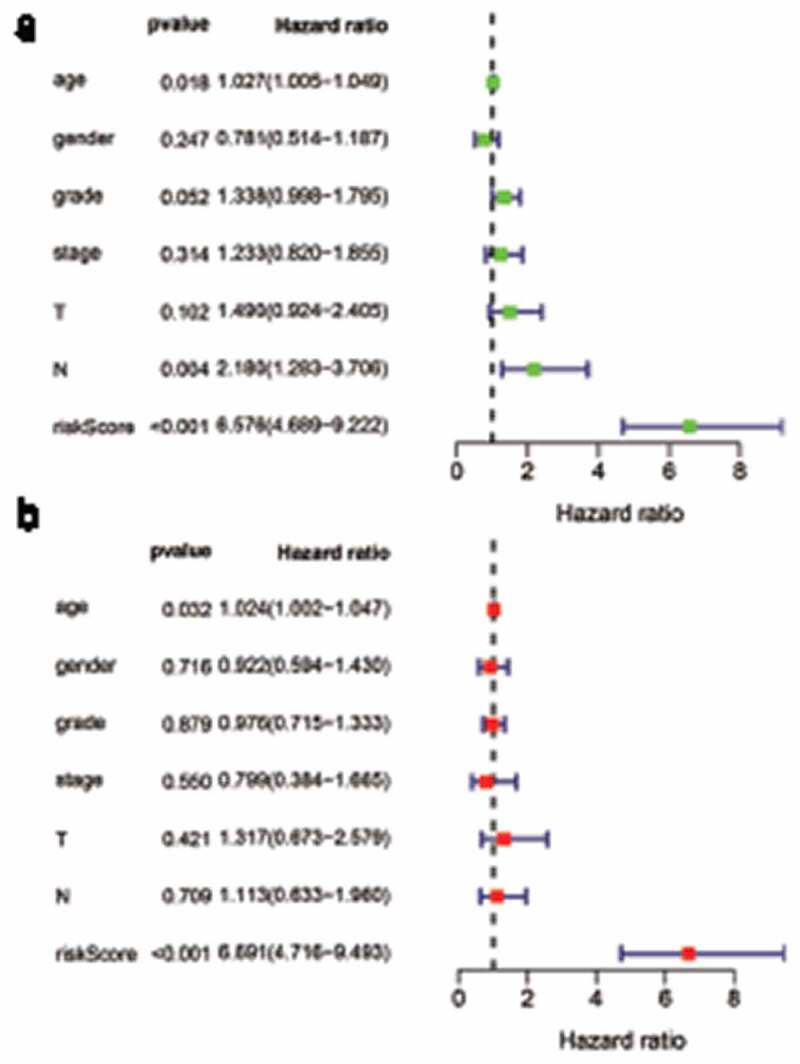
Univariate and multivariate analyses of prognostic factors. Clinical features such as age, sex, grade, stage, depth of tumor invasion (t) and lymph node metastasis (n) had no prognostic value in either the univariate or multivariate Cox analysis. Only the IRGPI signature remained an independent prognostic factor in the univariate and multivariate Cox analyses

The cohort containing TCGA (n = 490) gene expression data was used as the discovery cohort, and the genes with fantastically giant mutations were retained as candidates. The median absolute deviation (MAD > 0.5) was used for evaluation. The filtered discovery dataset was used to assemble a prognostic mannequin with the aid of the usage of 1,638 unique IRGs, which were bought from the ImmPort database and included 17 categories, and 578 IRGs were measured in the discovery set as well as on different platforms. Next, we identified IRGPs. After removing IRGPs with a small error (MAD < 0.5) and screening the survival of related patients (*p* < 0.001), the remaining IRGPs were chosen as preliminary candidate IRGPs. We then described the IRGPI and used Lasso Cox proportional hazards regression on the set to select 47 IRGPs for inclusion in our last hazard risk scoring model. Table S1 shows the information of 47 IRGPs included in the IRGPI. Then, the IRGPI was used to calculate the risk score for each patient in the group. According to an analysis of a ROC curve for survival, the optimal cutoff value for the IRGPI was 0.963 for patients divided into a high-risk group and a low-risk group (Figure 1). According to the cutoff value, OS can divide sufferers into low-risk groups and high-risk groups. The statistics confirmed that the OS of the high-risk group was extensively shorter than that of the low-risk group (*p* < 0.001) (Figure 2 and Table S2). To discover the prognostic capability of the IRGPI for different clinical factors, we conducted univariate and multivariate Cox proportional hazards regression analyses on the TCGA cohort. Clinical features such as age, sex, grade, stage, depth of tumor invasion (T) and lymph node metastasis (N) demonstrated no prognostic value in either the univariate or multivariate Cox analysis. Furthermore, in the univariate and multivariate Cox analyses, only IRGPI markers can still be used as an independent prognostic factor for pancreatic cancer (Figure 3(a,b)).

### Immune cell infiltration in different risk groups

**Figure 4.**
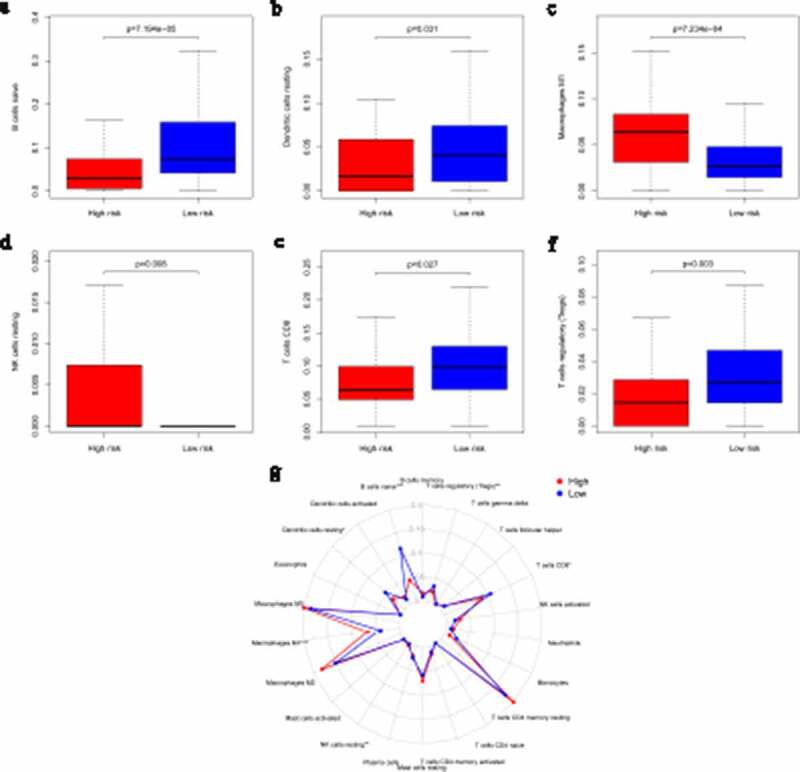
Immune infiltration statuses within IRGPI-defined risk groups. Summary of the abundances of 22 immune cell types estimated by CIBERSORT for different risk groups. (a–f) show that M1 macrophages and resting NK cells were significantly enriched in the high-risk group (M1 macrophages: *p* = 7.234e-4; resting NK cells: *p* = 0.008). The percentages of naïve B cells, resting dendritic cells, CD8 T cells and regulatory T cells (Tregs) were significantly higher in the low-risk group (naïve B cells: *p* = 7.194e-05; resting dendritic cells: *p* = 0.031; CD8 T cells: *p* = 0.027; Tregs: *p* = 0.003). A comparative summary of the CIBERSORT outputs for the high-risk group and low-risk group is shown in G

Many research have indicated that tumor-infiltrating immune cells are associated with tumor prognosis [20]. CIBERSORT has been used in previous tumor microenvironment-related reports to estimate immune cell subset frequencies [21,22]. CIBERSORT was used to estimate the relative proportions of 22 specific immune cell infiltrates in different risk groups of pancreatic patients. Our consequences confirmed that different immune cells were enriched in different risk groups. M1 macrophages and resting NK cells were significantly enriched in the high-risk group (M1 macrophages: *p* = 7.234e-4; resting NK cells: *p* = 0.008), while the percentages of naïve B cells, resting dendritic cells, CD8 T cells and regulatory T cells (Tregs) were significantly higher in the low-risk group (naïve B cells: *p* = 7.194e-05; resting dendritic cells: *p* = 0.031; CD8 T cells: *p* = 0.027; Tregs: *p* = 0.003; Figure 4(a–f), Table S3). A comparative summary of the CIBERSORT results for the high-risk group and low-risk group is shown in Figure 4(g).

**Figure 5.**
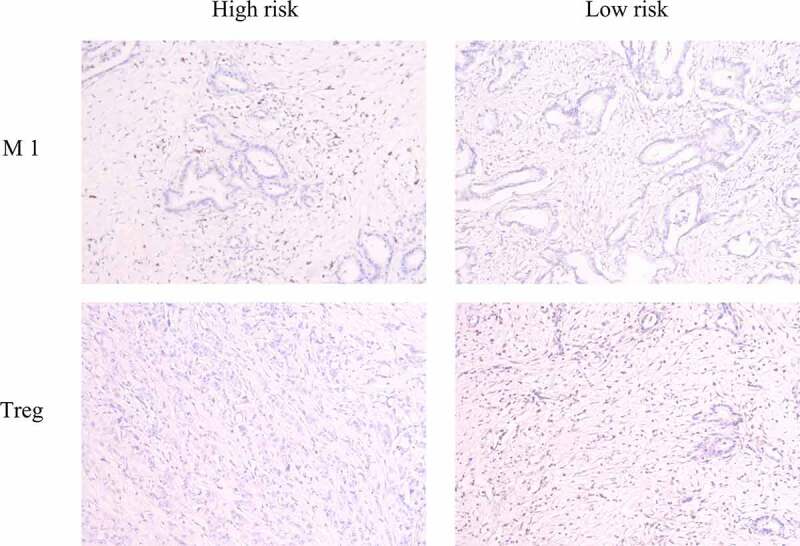
Immunohistochemical detection of immune cells. M1 macrophage levels were significantly higher in the high-risk group, while Treg levels were significantly higher in the low-risk group

We collected six pancreatic cancer tissues obtained from the Department of General Surgery between 2016 and 2017. Three of these patients died within 1 year of surgery, which we defined as the high-risk group. The remaining patients had a survival time of more than 1 year, which we defined as the low-risk group. We used an immunohistochemical method to detect the infiltration of M1 macrophages and Tregs by using specific markers (iNOS and FOXP3, respectively) [23]. The results showed that the M1 macrophage marker was significantly highly expressed in the high-risk group and that Treg levels were significantly higher in the low-risk group, which was consistent with our previous analysis (Figure 5).

### Functional assessment of the IRGPI signature

**Figure 6.**
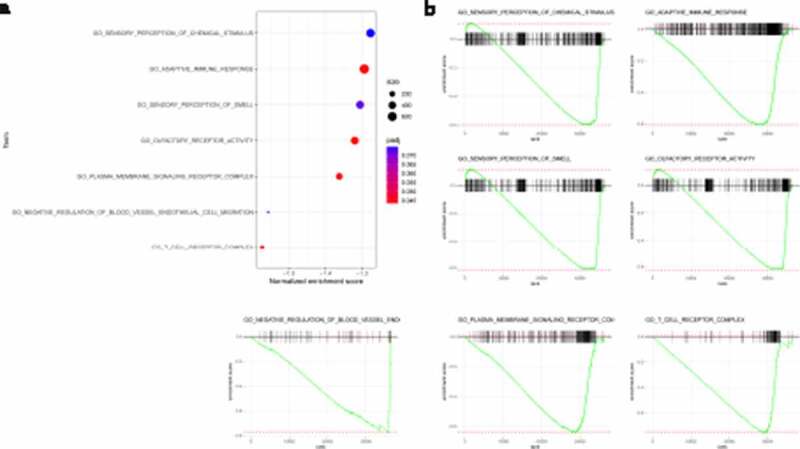
Immune-related signature gene analysis. Gene ontology (GO) analysis of the immune signature genes. GO analysis confirmed that the IRGPI signature genes in the cohort were mostly party to sensory perception of a chemical stimulus and the adaptive immune response

GO analysis and GSEA were used to discover the biological processes and signaling pathways altered in relation to the IRGPI signature. GO analysis confirmed that the IRGPI signature genes in the cohort were mostly party to sensory perception of a chemical stimulus and the adaptive immune response (Figure 6(a) and Table S4). GSEA was conducted to compare the high-risk and low-risk groups to investigate significantly altered pathways. This analysis confirmed that pathways related to sensory perception of a chemical stimulus and the adaptive immune response were significantly different (Figure 6(b)). These pathways provide a basis for studying the molecular mechanism of IRGPI signaling to predict the prognosis of pancreatic cancer.

## Discussion

Pancreatic cancer is an aggressive disease that frequently presents at an advanced stage [24]. Although much research has been performed, pancreatic cancer is still resistant to most available therapies [25]. In some recent studies, some gene alterations (KRAS, TP53, CDKN2A and SMAD4) have been shown to be associated with the OS of pancreatic cancer patients [26], but the proportion of pancreatic cancer patients with mutations in at least one of these four genes was found to be less than 40%[27]. The expression of other genes is less specific in pancreatic cancer and cannot be used as an independent prognostic indicator [28]. Therefore, the identification of a reliable prognostic biomarker to predict the survival rate of patients with serous pancreatic cancer is urgently needed.

Considering the inherent biological heterogeneity of tumors and the technical deviation caused by sequencing platforms, prognostic risk models need to correctly reflect the gene expression profile, which is a challenge in data analysis. To achieve robust prognostic prediction, we adopted a new data analysis method without taking into account the technology biases of different platforms [29].

In our study, IRGP signature was identified to predict the OS of pancreatic cancer patients. The prognostic signature consisted of 47 IRGPs, including many reportedly important genes. The CXC ligand family (CXCL5, CXCL9, CXCL10, CXCL11, and CXCL17) plays a vital role in regulating pancreatic cancer progression [30]. A recent study identified c-Met on circulating exosomes as a diagnostic and prognostic marker for pancreatic cancer [31]. ABCB2 (TAP1) is regarded as the downstream target of SHH signaling and plays an important role in enhancing the drug resistance of pancreatic ductal adenocarcinoma [32]. The interferon regulatory factor family [33] and some members of the interleukin family are indicators of prognosis in pancreatic cancer [34]. Then, we found that M1 macrophages and resting NK cells were significantly enriched in the high-risk group, while the percentages of naïve B cells, resting dendritic cells, CD8 T cells and Tregs were significantly higher in the low-risk group, and we verified these findings with immunohistochemical experiments. Previous studies have reported that pancreatic cancer in high-risk patients is characterized by an ‘immune-rich’ microenvironment with high estimated levels of M1 macrophages and low levels of Tregs [23,35], which is consistent with our results. GO analysis confirmed that the IRGPI signature genes in the cohort were mostly party to sensory perception of a chemical stimulus and the adaptive immune response. These pathways provide evidence for the molecular mechanism of IRGPI signaling and thus predict the prognosis of pancreatic cancer.

Currently, there are few studies on screening and identification of molecular markers to predict the prognosis of pancreatic cancer by detecting a large number of immunomodulatory gene expression profiles. There have been studies that have analyzed that an immune-related gene pairs signature predicts overall survival in colorectal cancer [36], serous ovarian carcinoma [15] and hepatocellular carcinoma [37]. But no one has analyzed it in pancreatic cancer. We systematically used public data to comprehensively analyze immune-regulated gene pair expression profiles and clinical data in pancreatic cancer. Our study efficiently built a robust IRGP signature with prognostic value for pancreatic cancer, presenting a conceivable method for deciding on a preoperative treatment.

There were some limitations to our study. Our prognostic signature consisted of 47 IRGPs, which is a large number. This might make it very difficult to apply the signature for clinical examination. The reason is that unlike other tumors, pancreatic cancer progresses rapidly with few specific symptoms, and its mechanism is elusive [24]. Our research is based on retrospective studies, and more clinical specimens and data are needed for further verification.

## Conclusion

We systematically used public data to comprehensively analyze IRGP expression profiles and clinical data of pancreatic cancer. And we efficiently built a robust IRGP signature with prognostic value for pancreatic cancer, providing a potential strategy for choosing a preoperative treatment. Our immune gene signature can effectively predict the survival of patients with Pancreatic cancer. In addition, the feature provides a panoramic view of the tumor immune microenvironment and will be a beneficial predictive tool to determine whether the certain patient could benefit from immunotherapy.

## Supplementary Material

Supplemental Material

## Highlights

1. In our study, IRGP signature was identified to predict the OS of pancreatic cancer patients.
2. We determined that immune genes are enriched in different risk groups.
3. We provide a useful predictive tool to determine the pancreatic cancer patients who is likely to benefit from immunotherapy.

## References

[1] Patel SH, Edwards MJ, Ahmad SA. Intracellular ion channels in pancreas cancer. Cell Physiol Biochem. 2019;53:44–51. 31834994 10.33594/000000193

[2] McGuire S. World cancer report 2014. Geneva, Switzerland: world Health Organization, International Agency for Research on Cancer, WHO Press, 2015. Advances in Nutrition (Bethesda, Md). 2016;7:418–419. 10.3945/an.116.012211 PMC4785485 26980827

[3] Siegel RL, Miller KD. Cancer statistics. CA Cancer J Clin. 2019;69(1):7–34. doi: 10.3322/caac.21551. 30620402

[4] Andersen DK, Korc M, Petersen GM, et al. Diabetes, pancreatogenic diabetes, and pancreatic cancer. Diabetes. 2017 May;66(5):1103-1110 10.2337/db16-1477 PMC5399609 28507210

[5] Bosetti C, Rosato V, Li D, *et al*. Diabetes, antidiabetic medications, and pancreatic cancer risk: an analysis from the international pancreatic cancer case-control consortium. Ann Oncol. 2014;25:2065–2072. 25057164 10.1093/annonc/mdu276 PMC4176453

[6] Strobel O. Optimizing the outcomes of pancreatic cancer surgery. Neoptolemos J. 2019;16:11–26. 10.1038/s41571-018-0112-1 30341417

[7] Yamashita Y, Kitano M. Value of endoscopy for early diagnosis of pancreatic carcinoma. Dig Endosc. Dig Endosc. 2020 Jan;32(1):27-36 .doi:10.1111/den.13467. 31219200

[8] Siegel RL, Miller KD, Jemal A. Cancer statistics.CA Cancer J Clin. 2018 Jan;68(1):7-30. 10.3322/caac.21442 29313949

[9] Luo G, Fan Z, Gong Y, et al. Characteristics and outcomes of pancreatic cancer by histological subtypes. Pancreas. 2019;48:817–822. 31210663 10.1097/MPA.0000000000001338

[10] Yako YY, Kruger D, Smith M, et al. Cytokines as biomarkers of pancreatic ductal adenocarcinoma: a systematic review. PloS One. 2016;11:e0154016. 27170998 10.1371/journal.pone.0154016 PMC4865360

[11] Lu C, Paschall AV, Shi H, et al. The MLL1-H3K4me3 Axis-Mediated PD-L1 expression and pancreatic cancer immune evasion. J Natl Cancer Inst. 2017; (109):djw283. DOI:10.1093/jnci/djw283. PMC5291187 28131992

[12] Topalian SL, Hodi FS, Brahmer JR, et al. Safety, activity, and immune correlates of anti-PD-1 antibody in cancer. N Engl J Med. 2012;366:2443–2454. 22658127 10.1056/NEJMoa1200690 PMC3544539

[13] Pihlak R, Weaver JMJ, Valle JW, et al. Advances in molecular profiling and categorisation of pancreatic adenocarcinoma and the implications for therapy. Cancers (Basel). 2018 Jan 12;10(1):17. doi: 10.3390/cancers10010017 PMC5789367 29329208

[14] Leek JT, Scharpf RB, Bravo HC, et al. Tackling the widespread and critical impact of batch effects in high-throughput data. Nat Rev Genet. 2010;11:733–739. 20838408 10.1038/nrg2825 PMC3880143

[15] Zhang L, Zhu P, Tong Y, et al. An immune-related gene pairs signature predicts overall survival in serous ovarian carcinoma. Oncotargets and Therapy. 2019;12:7005–7014. 31695415 10.2147/OTT.S200191 PMC6718165

[16] Heinäniemi M, Nykter M, Kramer R, et al. Gene-pair expression signatures reveal lineage control. Nat Methods. 2013;10:577–583. 23603899 10.1038/nmeth.2445 PMC4131748

[17] Li B, Cui Y, Diehn M, et al. Development and validation of an individualized immune prognostic signature in early-stage nonsquamous non-small cell lung cancer. JAMA Oncol. 2017;3:1529–1537. 28687838 10.1001/jamaoncol.2017.1609 PMC5710196

[18] Popovici V, Budinska E, Tejpar S, et al. Identification of a poor-prognosis BRAF-mutant-like population of patients with colon cancer. J Clin Oncol. 2012;30:1288–1295. 22393095 10.1200/JCO.2011.39.5814

[19] Padoan A, Plebani M, Basso D. Inflammation and pancreatic cancer: focus on metabolism, cytokines, and immunity. Int J Mol Sci. 2019;20(3):676. 10.3390/ijms20030676 PMC6387440 30764482

[20] Liu X, Wu S, Yang Y, et al. The prognostic landscape of tumor-infiltrating immune cell and immunomodulators in lung cancer. Biomed Pharmacothe. 2017;95:55–61. 10.1016/j.biopha.2017.08.003 28826097

[21] Cole C, Lau S, Backen A, et al. Inhibition of FGFR2 and FGFR1 increases cisplatin sensitivity in ovarian cancer. Cancer Biol Ther. 2010;10:495–504. 20595807 10.4161/cbt.10.5.12585 PMC3040972

[22] Motoyama K, Tanaka F, Kosaka Y, et al. Clinical significance of BMP7 in human colorectal cancer. Ann Surg Oncol. 2008;15:1530–1537. 18259822 10.1245/s10434-007-9746-4

[23] Karamitopoulou E. Tumour microenvironment of pancreatic cancer: immune landscape is dictated by molecular and histopathological features. Br J Cancer. 2019;121:5–14. 31110329 10.1038/s41416-019-0479-5 PMC6738327

[24] Hidalgo M, Cascinu S, Kleeff J, et al. Addressing the challenges of pancreatic cancer: future directions for improving outcomes. Pancreatology. 2015;15:8–18. 25547205 10.1016/j.pan.2014.10.001

[25] Neoptolemos JP, Palmer DH, Ghaneh P, et al. Comparison of adjuvant gemcitabine and capecitabine with gemcitabine monotherapy in patients with resected pancreatic cancer (ESPAC-4): a multicentre, open-label, randomised, phase 3 trial. Lancet. 2017;389:1011–1024. 28129987 10.1016/S0140-6736(16)32409-6

[26] Qian ZR, Rubinson DA, Nowak JA, et al. Association of alterations in main driver genes with outcomes of patients with resected pancreatic ductal adenocarcinoma. JAMA Oncol. 2018;4:e173420. 29098284 10.1001/jamaoncol.2017.3420 PMC5844844

[27] Yachida S, White CM, Naito Y, et al. Clinical significance of the genetic landscape of pancreatic cancer and implications for identification of potential long-term survivors. Clin Cancer Res off J Am Assoc Cancer Res. 2012;18:6339–6347. 10.1158/1078-0432.CCR-12-1215 PMC3500447 22991414

[28] Bailey P, Chang DK, Nones K, et al. Genomic analyses identify molecular subtypes of pancreatic cancer. Nature. 2016;531:47–52. 26909576 10.1038/nature16965

[29] Eddy JA, Sung J, Geman D, et al. Relative expression analysis for molecular cancer diagnosis and prognosis. Technol Cancer Res Treat. 2010;9:149–159. 20218737 10.1177/153303461000900204 PMC2921829

[30] Lee NH, Nikfarjam M, He H. Functions of the CXC ligand family in the pancreatic tumor microenvironment. Pancreatology. 2018;18:705–716. 30078614 10.1016/j.pan.2018.07.011

[31] Lux A, Kahlert C, Grützmann R, et al. c-Met and PD-L1 on circulating exosomes as diagnostic and prognostic markers for pancreatic cancer. 2019;20(13):3305. 10.3390/ijms20133305 PMC6651266 31284422

[32] Xu M, Li L, Liu Z, et al. ABCB2 (TAP1) as the downstream target of SHH signaling enhances pancreatic ductal adenocarcinoma drug resistance. Cancer Lett. 2013;333:152–158. 23340176 10.1016/j.canlet.2013.01.002

[33] Sakai T, Mashima H, Yamada Y, et al. The roles of interferon regulatory factors 1 and 2 in the progression of human pancreatic cancer. Pancreas. 2014;43:909–916. 24632547 10.1097/MPA.0000000000000116

[34] Feng L, Qi Q, Wang P, et al. Serum levels of IL-6, IL-8, and IL-10 are indicators of prognosis in pancreatic cancer. J Int Med Res. 2018;46:5228–5236. 30304975 10.1177/0300060518800588 PMC6300928

[35] Wartenberg M, Cibin S, Zlobec I, et al. Integrated genomic and immunophenotypic classification of pancreatic cancer reveals three distinct subtypes with prognostic/predictive significance. Clin Cancer Res. 2018;24:4444–4454. 10.1158/1078-0432.CCR-17-3401 29661773

[36] Wu J, Zhao Y, Zhang J, et al. Development and validation of an immune-related gene pairs signature in colorectal cancer. Oncoimmunology. 2019;8:1596715. 31143520 10.1080/2162402X.2019.1596715 PMC6527298

[37] Sun XY, Yu SZ, Zhang HP, et al. A signature of 33 immune-related gene pairs predicts clinical outcome in hepatocellular carcinoma. Cancer Med. 2020;9:2868–2878. 32068352 10.1002/cam4.2921 PMC7163092

