# Supplementary material for: Individualized prognostic signature for pancreatic carcinoma validated by integrating immune-related gene pairs (IRGPs)
Source: Bioengineered. 2021 Jan 4;12(1):88–95. doi: 10.1080/21655979.2020.1860493 (PMC8806356; doi:10.1080/21655979.2020.1860493)
Supplement: Supplemental Material [file KBIE_A_1860493_SM3306.zip › supplementary/highlights.docx]

**Highlights**

1. In our study, IRGP signature was identified to predict the OS of pancreatic cancer patients.
2. We determined that immune genes are enriched in different risk groups.
3. We provide a useful predictive tool to determine the pancreatic cancer patients who is likely to benefit from immunotherapy.
