# Supplementary material for: Individualized prognostic signature for pancreatic carcinoma validated by integrating immune-related gene pairs (IRGPs)
Source: Bioengineered. 2021 Jan 4;12(1):88–95. doi: 10.1080/21655979.2020.1860493 (PMC8806356; doi:10.1080/21655979.2020.1860493)
Supplement: Supplemental Material [file KBIE_A_1860493_SM3306.zip › supplementary/supplement table.docx]

Table S1 Model information about IRGPI

| **IRG 1** | **Immune processes** | **IRG 2** | **Immune processes** | **Coefficient** |
| --- | --- | --- | --- | --- |
| CD1D | Antigen_Processing_and_Presentation | DKK1 | Cytokines | -0.34 |
| HLA-H | Antigen_Processing_and_Presentation | TAP1 | Antigen_Processing_and_Presentation | -0.34 |
| HSPA6 | Antigen_Processing_and_Presentation | IL20RB | Cytokine_Receptors | -0.10 |
| ERAP2 | Antigen_Processing_and_Presentation | NR2F1 | Cytokine_Receptors | 0.11 |
| ERAP2 | Antigen_Processing_and_Presentation | SSTR1 | Cytokine_Receptors | 0.45 |
| CXCL10 | Antimicrobials | NFKBIE | BCRSignalingPathway | 0.17 |
| CXCL10 | Antimicrobials | SLIT2 | Chemokines | 0.20 |
| CXCL9 | Cytokines | HCST | NaturalKiller_Cell_Cytotoxicity | 0.07 |
| CXCL5 | Antimicrobials | IFIH1 | Antimicrobials | 0.16 |
| CXCL5 | Antimicrobials | VDR | Antimicrobials | 0.05 |
| CXCL5 | Antimicrobials | SCGB3A1 | Cytokines | 0.02 |
| CXCL11 | Antimicrobials | CD79A | BCRSignalingPathway | 0.01 |
| CXCL11 | Antimicrobials | LILRB3 | BCRSignalingPathway | 0.34 |
| DEFB1 | Chemokines | SLC40A1 | Antimicrobials | 0.16 |
| TGFB1 | Cytokines | MET | Cytokine_Receptors | -0.00 |
| APOBEC3G | Antimicrobials | PLXNC1 | Chemokine_Receptors | 0.59 |
| IRF3 | Antimicrobials | CKLF | Chemokines | -0.22 |
| IRF3 | Antimicrobials | NAMPT | Cytokines | -0.07 |
| RBP5 | Antimicrobials | LGR6 | Cytokine_Receptors | -0.10 |
| FABP5 | Antimicrobials | PDGFC | Cytokines | -0.15 |
| IDO1 | Antimicrobials | MAP3K14 | TCRsignalingPathway | 0.12 |
| RARRES3 | Antimicrobials | JUND | Antimicrobials | 0.07 |
| GRN | Cytokines | SPP1 | Cytokines | -0.44 |
| PDCD1 | TCRsignalingPathway | GHRL | Cytokines | 0.02 |
| ANXA6 | Antimicrobials | RAC2 | BCRSignalingPathway | -0.10 |
| CCR7 | Chemokine_Receptors | FGF7 | Cytokines | -0.57 |
| NRAS | NaturalKiller_Cell_Cytotoxicity | AKT1 | TCRsignalingPathway | 0.01 |
| IGKV1-5 | BCRSignalingPathway | IL1RN | Cytokines | -0.17 |
| CXCL17 | Chemokines | IL4R | Cytokine_Receptors | 0.08 |
| EDN2 | Chemokines | NGFR | Cytokine_Receptors | 0.32 |
| SEMA3C | Chemokines | PLXNB1 | Chemokine_Receptors | 0.09 |
| SEMA3C | Chemokines | OGFR | Cytokine_Receptors | 0.01 |
| PLAUR | Cytokine_Receptors | MET | Cytokine_Receptors | -0.23 |
| PLXNB1 | Cytokine_Receptors | MET | Cytokine_Receptors | -0.25 |
| AREG | Cytokines | FYN | NaturalKiller_Cell_Cytotoxicity | 0.19 |
| CHGA | Cytokines | TGFB3 | Cytokines | -0.01 |
| CHGA | Cytokines | IL22RA1 | Cytokine_Receptors | -0.06 |
| CHGA | Cytokines | NR4A2 | Cytokine_Receptors | -0.02 |
| FGF7 | Cytokines | GHRL | Cytokines | 0.12 |
| HBEGF | Cytokines | IL3RA | Cytokine_Receptors | 0.00 |
| UCN | Cytokines | IL20RB | Cytokine_Receptors | -0.00 |
| CRLF3 | Cytokine_Receptors | IL20RB | Cytokine_Receptors | -0.19 |
| IL10RA | Cytokine_Receptors | FAS | NaturalKiller_Cell_Cytotoxicity | -0.03 |
| IL1R2 | Cytokine_Receptors | HCST | NaturalKiller_Cell_Cytotoxicity | 0.07 |
| IL20RB | Cytokine_Receptors | TNFRSF4 | Cytokine_Receptors | 0.02 |
| IL22RA1 | Interleukins_Receptor | ITGB2 | NaturalKiller_Cell_Cytotoxicity | 0.23 |

Table S2. Patients immune risk stratification

| **ID** | **Risk** | **RiskScore** |
| --- | --- | --- |
| TCGA-IB-AAUQ | high | 1.621033 |
| TCGA-HZ-A9TJ | low | -0.56441 |
| TCGA-H6-8124 | low | 0.746622 |
| TCGA-HV-A5A4 | low | -2.35088 |
| TCGA-2J-AAB6 | high | 1.731176 |
| TCGA-3E-AAAY | low | -1.55 |
| TCGA-F2-7276 | low | 0.759498 |
| TCGA-US-A77J | low | -0.9146 |
| TCGA-3A-A9I9 | low | -0.09387 |
| TCGA-3A-A9IU | low | 0.741936 |
| TCGA-US-A776 | low | -1.49844 |
| TCGA-IB-7654 | low | 0.070044 |
| TCGA-2J-AABT | low | -1.28503 |
| TCGA-2L-AAQA | high | 1.734816 |
| TCGA-LB-A9Q5 | high | 1.132101 |
| TCGA-2L-AAQL | low | -0.35697 |
| TCGA-HZ-7926 | low | 0.899375 |
| TCGA-IB-7893 | high | 1.700532 |
| TCGA-FB-AAQ2 | low | 0.901385 |
| TCGA-IB-7652 | low | -0.2462 |
| TCGA-IB-7891 | low | 0.315722 |
| TCGA-HZ-7920 | low | 0.294746 |
| TCGA-IB-AAUW | low | 0.328521 |
| TCGA-L1-A7W4 | high | 1.085953 |
| TCGA-XD-AAUH | low | -2.89763 |
| TCGA-HZ-8519 | low | -0.87232 |
| TCGA-2J-AABF | low | 0.484256 |
| TCGA-IB-7888 | low | -0.98705 |
| TCGA-HZ-7918 | low | -0.37566 |
| TCGA-RB-AA9M | low | -1.41707 |
| TCGA-2L-AAQJ | low | 0.931681 |
| TCGA-FB-A545 | low | -0.40756 |
| TCGA-3A-A9IN | low | -3.22049 |
| TCGA-HZ-A49H | low | -0.69346 |
| TCGA-HZ-A8P1 | low | -0.91299 |
| TCGA-HZ-8002 | low | 0.102193 |
| TCGA-FB-AAPP | low | 0.369953 |
| TCGA-IB-A7LX | high | 2.316181 |
| TCGA-HZ-8637 | low | -0.56369 |
| TCGA-IB-AAUT | low | -1.58058 |
| TCGA-IB-7646 | high | 2.03211 |
| TCGA-2J-AABA | low | 0.009155 |
| TCGA-HV-AA8X | low | 0.963432 |
| TCGA-IB-A5SQ | low | -0.03746 |
| TCGA-YY-A8LH | low | -0.6897 |
| TCGA-FB-A4P6 | low | -1.1775 |
| TCGA-HZ-A8P0 | low | -1.1391 |
| TCGA-IB-AAUN | high | 2.12097 |
| TCGA-US-A77E | high | 1.256279 |
| TCGA-FB-AAPZ | low | -0.24906 |
| TCGA-LB-A7SX | high | 1.191969 |
| TCGA-HZ-8638 | high | 1.590093 |
| TCGA-XN-A8T5 | low | -1.23706 |
| TCGA-IB-8127 | low | -0.14623 |
| TCGA-HZ-A77P | low | -0.23684 |
| TCGA-H8-A6C1 | low | -1.29401 |
| TCGA-2J-AABE | low | -1.00946 |
| TCGA-3A-A9IX | low | -1.48424 |
| TCGA-HZ-7289 | low | 0.097053 |
| TCGA-HV-A7OP | low | -1.37203 |
| TCGA-2J-AABK | low | 0.282704 |
| TCGA-2J-AAB9 | low | -0.29108 |
| TCGA-IB-7887 | high | 1.927869 |
| TCGA-HZ-A4BH | low | 0.163898 |
| TCGA-2J-AABU | high | 1.754453 |
| TCGA-HZ-A77O | high | 1.624828 |
| TCGA-Q3-A5QY | low | -0.70653 |
| TCGA-HZ-8005 | high | 2.578231 |
| TCGA-2L-AAQI | high | 2.377822 |
| TCGA-YB-A89D | low | 0.02899 |
| TCGA-3A-A9IJ | low | -3.34243 |
| TCGA-US-A779 | low | 0.203809 |
| TCGA-3A-A9J0 | low | -0.9814 |
| TCGA-IB-AAUU | low | -0.62101 |
| TCGA-2L-AAQE | low | 0.368437 |
| TCGA-PZ-A5RE | low | 0.420391 |
| TCGA-3A-A9IL | low | -3.28852 |
| TCGA-IB-7890 | low | 0.629274 |
| TCGA-XD-AAUG | low | -1.80698 |
| TCGA-IB-7649 | low | -0.03434 |
| TCGA-IB-A6UG | low | 0.634151 |
| TCGA-3A-A9IB | high | 1.622268 |
| TCGA-HZ-7923 | low | -1.59756 |
| TCGA-FB-A7DR | high | 1.255018 |
| TCGA-F2-6880 | low | -0.44267 |
| TCGA-2J-AAB1 | high | 2.507839 |
| TCGA-M8-A5N4 | low | 0.405055 |
| TCGA-2J-AABI | low | -0.62538 |
| TCGA-IB-7885 | low | -0.42091 |
| TCGA-HV-A5A5 | low | -0.77252 |
| TCGA-IB-A5ST | low | -1.80384 |
| TCGA-F2-7273 | low | -0.06192 |
| TCGA-FB-AAPS | low | -1.08218 |
| TCGA-IB-A7M4 | low | 0.813289 |
| TCGA-FB-A78T | low | 0.311995 |
| TCGA-HZ-A49G | low | -1.12256 |
| TCGA-IB-AAUM | low | -0.82657 |
| TCGA-FB-AAPU | low | -0.12387 |
| TCGA-IB-AAUR | low | -1.04319 |
| TCGA-S4-A8RM | low | -0.19749 |
| TCGA-HZ-7925 | low | 0.139333 |
| TCGA-HZ-8636 | low | 0.711809 |
| TCGA-HV-A7OL | low | -0.08757 |
| TCGA-RB-A7B8 | low | 0.155705 |
| TCGA-US-A77G | high | 1.017177 |
| TCGA-HZ-A49I | low | -0.82696 |
| TCGA-IB-A5SO | low | -0.18864 |
| TCGA-HV-A5A3 | high | 1.340238 |
| TCGA-IB-A5SP | low | -0.06748 |
| TCGA-3A-A9IH | low | -0.22087 |
| TCGA-HZ-A77Q | low | -0.24499 |
| TCGA-FB-AAQ1 | high | 1.093904 |
| TCGA-IB-7651 | low | 0.506096 |
| TCGA-XD-AAUI | low | 0.74119 |
| TCGA-2L-AAQM | low | -2.88981 |
| TCGA-IB-AAUS | low | -0.75701 |
| TCGA-FB-AAQ3 | high | 3.230353 |
| TCGA-OE-A75W | high | 1.328435 |
| TCGA-XN-A8T3 | low | -0.70405 |
| TCGA-IB-7645 | low | -1.48145 |
| TCGA-3A-A9IO | low | -3.13564 |
| TCGA-3A-A9IV | low | -2.80303 |
| TCGA-RL-AAAS | low | -0.64248 |
| TCGA-F2-A44G | high | 1.282903 |
| TCGA-Z5-AAPL | low | -0.39121 |
| TCGA-3E-AAAZ | low | -1.20177 |
| TCGA-HZ-8003 | low | 0.21216 |
| TCGA-2J-AABR | low | 0.246616 |
| TCGA-HZ-8001 | low | -1.0194 |
| TCGA-IB-7889 | low | 0.940031 |
| TCGA-FB-A5VM | low | -0.21547 |
| TCGA-HZ-7919 | high | 1.27893 |
| TCGA-3A-A9IZ | high | 1.746107 |
| TCGA-2J-AAB8 | low | 0.193384 |
| TCGA-3A-A9I5 | low | -2.01281 |
| TCGA-2J-AABH | low | -0.80265 |
| TCGA-IB-A6UF | low | 0.157167 |
| TCGA-HZ-A4BK | low | -1.23256 |
| TCGA-S4-A8RO | low | 0.113186 |
| TCGA-F2-A7TX | high | 2.020971 |
| TCGA-3A-A9IC | low | -0.47077 |
| TCGA-IB-AAUO | high | 2.092976 |
| TCGA-IB-AAUV | low | -1.40785 |
| TCGA-F2-6879 | high | 1.587456 |
| TCGA-IB-7897 | low | -0.10318 |
| TCGA-HV-AA8V | low | -0.60847 |
| TCGA-IB-AAUP | low | -1.50968 |
| TCGA-XD-AAUL | low | -0.47869 |
| TCGA-S4-A8RP | low | -0.75325 |
| TCGA-HZ-8317 | high | 1.158983 |
| TCGA-IB-A5SS | high | 1.437889 |
| TCGA-IB-7644 | low | 0.88978 |
| TCGA-FB-A4P5 | low | 0.125311 |
| TCGA-2J-AAB4 | low | -0.21253 |
| TCGA-FB-AAPQ | low | -0.21526 |
| TCGA-H6-A45N | low | -0.77676 |
| TCGA-Q3-AA2A | low | 0.083163 |
| TCGA-2J-AABP | low | -1.01762 |
| TCGA-3A-A9I7 | low | -1.29942 |
| TCGA-FB-AAPY | low | -0.15949 |
| TCGA-F2-A44H | low | -1.07262 |
| TCGA-YH-A8SY | low | 0.524735 |
| TCGA-HV-A5A6 | low | -0.89008 |
| TCGA-3A-A9IR | low | -2.83871 |
| TCGA-FB-AAQ6 | high | 1.542907 |
| TCGA-IB-7886 | high | 1.835224 |
| TCGA-US-A774 | low | 0.441888 |
| TCGA-F2-A8YN | low | -0.18506 |
| TCGA-HZ-7922 | high | 1.058537 |
| TCGA-FB-AAQ0 | low | 0.962616 |
| TCGA-IB-8126 | low | 0.094604 |
| TCGA-3A-A9IS | low | -2.71812 |
| TCGA-2J-AABV | low | 0.080845 |
| TCGA-HZ-8315 | high | 1.834264 |
| TCGA-2J-AABO | low | -0.57442 |
| TCGA-LB-A8F3 | low | -0.23681 |
| TCGA-HZ-7924 | low | -0.51971 |

Table S3. Immune cells infiltration within different risk groups

| **Cell** | ***p-*value** | **Sig** |
| --- | --- | --- |
| B cells naïve | 7.19E-05 | *** |
| T cells CD8 | 0.027 | * |
| T cells regulatory (Tregs) | 0.003 | ** |
| NK cells resting | 0.008 | ** |
| Macrophages M1 | 0.001 | *** |
| Dendritic cells resting | 0.031 | * |

Table S4. GSEA

| **Pathway** | **ES** | **NES** | ***p*-value** | **Adjusted *p*-value** |
| --- | --- | --- | --- | --- |
| GO_T_CELL_RECEPTOR_COMPLEX | -0.756511731 | -1.58 | 1.02E-05 | 0.04 |
| GO_ADAPTIVE_IMMUNE_RESPONSE | -0.60653132 | -1.30 | 2.00E-05 | 0.04 |
| GO_OLFACTORY_RECEPTOR_ACTIVITY | -0.620802829 | -1.32 | 3.00E-05 | 0.04 |
| GO_PLASMA_MEMBRANE_SIGNALING_RECEPTOR_COMPLEX | -0.643155374 | -1.36 | 3.00E-05 | 0.04 |
| GO_SENSORY_PERCEPTION_OF_SMELL | -0.613709938 | -1.31 | 7.00E-05 | 0.07 |
| GO_NEGATIVE_REGULATION_OF_BLOOD_VESSEL_ENDOTHELIAL_CELL_MIGRATION | -0.776547273 | -1.58 | 7.43E-05 | 0.07 |
| GO_SENSORY_PERCEPTION_OF_CHEMICAL_STIMULUS | -0.599593511 | -1.28 | 9.00E-05 | 0.07 |
