# Supplementary figures and images for: Individualized prognostic signature for pancreatic carcinoma validated by integrating immune-related gene pairs (IRGPs)
Source: Bioengineered. 2021 Jan 4;12(1):88–95. doi: 10.1080/21655979.2020.1860493 (PMC8806356; doi:10.1080/21655979.2020.1860493)

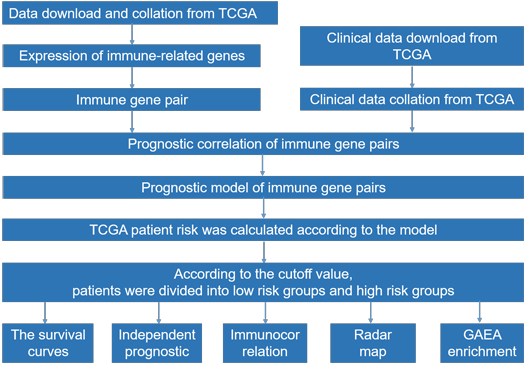

Supplement: Supplemental Material [file KBIE_A_1860493_SM3306.zip › supplementary/GraphicalAbstract.tif]
